# Dermatological Manifestations in the Intensive Care Unit: A Practical Approach

**DOI:** 10.1155/2020/9729814

**Published:** 2020-09-26

**Authors:** Mariona Badia, José Manuel Casanova, Lluís Serviá, Neus Montserrat, Jordi Codina, Javier Trujillano

**Affiliations:** ^1^Department of Intensive Care Medicine, Arnau de Vilanova Hospital, Lleida, Spain; ^2^Department of Dermatology, Arnau de Vilanova Hospital, IRBLLEIDA, Lleida, Spain

## Abstract

Dermatological problems are not usually related to intensive medicine because they are considered to have a low impact on the evolution of critical patients. Despite this, dermatological manifestations (DMs) are relatively frequent in critically ill patients. In rare cases, DMs will be the main diagnosis and will require intensive treatment due to acute skin failure. In contrast, DMs can be a reflection of underlying systemic diseases, and their identification may be key to their diagnosis. On other occasions, DMs are lesions that appear in the evolution of critical patients and are due to factors derived from the stay or intensive treatment. Lastly, DMs can accompany patients and must be taken into account in the comprehensive pathology management. Several factors must be considered when addressing DMs: on the one hand, the moment of appearance, morphology, location, and associated treatment and, on the other hand, aetiopathogenesis and classification of the cutaneous lesion. DMs can be classified into 4 groups: life-threatening DMs (uncommon but compromise the patient's life); DMs associated with systemic diseases where skin lesions accompany the pathology that requires admission to the intensive care unit (ICU); DMs secondary to the management of the critical patient that considers the cutaneous manifestations that appear in the evolution mainly of infectious or allergic origin; and DMs previously present in the patient and unrelated to the critical process. This review provides a characterization of DMs in ICU patients to establish a better identification and classification and to understand their interrelation with critical illnesses.

## 1. Introduction

Dermatological problems have received limited attention within the field of intensive care medicine, probably because they are considered within a specialty with a large outpatient component and rarely are serious and because of the limited impact they have on the prognosis of critical patients. Less than 0.5% of patients requiring admission to an intensive care unit (ICU) do so because of a primary dermatological problem [1]. However, and more generally, dermatological manifestations (DMs) in critical patients are relatively frequent and can be markers of extracutaneous pathology. In a previous study, we observed that 10.4% of patients admitted to an ICU had some type of DM [2], and a study by Agrawal observed DMs in up to 42.2% of critically ill patients [3]. Identifying these manifestations can be key in the recognition of an underlying disease. Examples include skin manifestations in meningococcal sepsis, skin as an expression of disseminated candidiasis, or ecthyma gangrenosum in *Pseudomonas aeruginosa* infection [4, 5]. Other times, DMs reflect secondary effects of drugs or devices [6, 7], problems derived from procedures [8], or simply a patient's critical situation [9, 10]. DMs can be presented in a highly varied way, and a definitive diagnosis often requires the collaboration of a dermatologist.

The objective of this review is to describe the most frequent DMs found in critically ill patients. Thermal injuries and necrotizing fasciitis, which constitute differentiated entities, are not included. This review consists of several sections: we will analyse the differentiating characteristics and the classification of DMs in critical patients, review published series, describe schematically the different types of DMs, and close with the conclusions.

### 1.1. Differentiating Characteristics of Dermatological Manifestations in Critical Patients

The management of critical patients requires a multidisciplinary evaluation and management as a fundamental part of their treatment. Rarely are patients admitted due to a dermatological disease; however, the DMs that appear during the evolution of the patients and that require treatment are relatively frequent [11]. Prolonged immobilization, insufficient nutritional support, impaired tissue perfusion, changes in body temperature, hygiene challenges, use of multiple drugs, and invasive processes may facilitate the development of DMs in ICU patients [12].

To these circumstances, we must add the lack of symptoms due to an altered state of consciousness, either by the use of sedative drugs or by neurological impairment, which makes communication with the patients difficult. There is little literature that specifically addresses DMs in the critical patient context, and there is no homogenous classification available.

Patients respond to the aggression of critical illnesses with profound changes at the immunological, endocrine, and vascular levels that results in impairments in tissue perfusion and changes in the microcirculation of the skin that alter the signs and symptoms of cutaneous lesions [13].

DMs that can be identified in Intensive Care Medicine have been classified in different ways. In some cases, a classification based on morphology or lesions [14] has been used according to whether the DM required the care of a dermatologist or only an intensivist [15]. We believe, concurring with other authors, that the classification that should be used in critical patients must have a dermatological base, but it should also include groups with differentiating characteristics in the diagnosis, treatment, and evolution during admission to the ICU [16–18].

In Table 1, a classification is proposed that includes the particularities of ICU patients. There are 4 main groups: life-threatening DMs (corresponding in the literature with the term primary lesions); DMs associated with systemic diseases (may indicate an underlying disease); DMs secondary to the management of the critical patient (divided into infectious, drug reactions, and secondary to devices); and previous DMs (named coincidental in previous studies [16, 17]) that, if not identified, could hinder or confuse the main diagnosis of the patient.

### 1.2. Review of Published Series on Dermatological Manifestations in the ICU

Table 2 shows the published works that include the follow-up of series of patients admitted to the ICU where the main DMs are analysed. Some studies focus on DMs associated with ICU treatment [15, 19]; others, such as studies on paediatric patients, include a morphological classification attributable to the importance of exanthematic childhood diseases.

It is difficult to compare different publications due to the lack of homogeneity in the DM classification; therefore, we have adapted the results obtained to the classification devised by Dunnil et al. [16] in a classic study that, with some modifications, has been maintained in our publications and in those made by Awal et al. [17, 18]. The advantage offered by this classification is that it groups DMs based on the situation of the critical patients without ignoring the origin of the DMs. Different studies show that dermatological admission is infrequent but that DMs usually accompany the evolution of critical patients either in the context of a systemic disease or adding to their main pathological process, influencing stay, and mortality.

The differences attributed to the country of origin of the different articles determine the type of lesions identified, as is the case in the study by Prashanth and Pai, in which 64% of patients had Dengue haemorrhagic fever [20]. We also verified the importance of the year of publication of the study. In our case, we went from 12 patients with DMs secondary to meningococcal meningitis in 1999 to 1 case in 2013, a situation that we attribute to the broad use of a meningococcal vaccine [2].

As limitations of the different studies, except for George [1], the studies were carried out in a single centre and were mainly retrospective, which hinders the generalization of the results. Furthermore, the type of DM studied differs among the authors. Some studies included necrotizing fasciitis [1] or pressure ulcers [18], which we exclude from classification because they have very well-defined characteristics and are treated independently in most cases.

All the studies described collaboration with a dermatologist specialist, either as a principal physician or as a consultant.

## 2. Classification of Dermatological Manifestations in Intensive Medicine

### 2.1. Life-Threatening Dermatological Manifestations

These DMs require admission to the ICU. They are rare (as seen in Table 2) but severe and are associated with high mortality. They cause loss or deterioration of cutaneous functions, leading to an imbalance and failure of the cutaneous barrier, defined as acute skin failure [21]. Destruction of the stratum corneum, the outermost layer of the epidermis, causes the loss of fluids, proteins, and electrolytes, immunological dysfunction with the consequent risk of infection, thermoregulatory impairment, and a hypercatabolic state.

Within this group, we include various entities such as immunobullous diseases, generalized pustular psoriasis, erythroderma, severe cutaneous adverse reactions (SCARs), and infectious diseases such as scalded skin syndrome.

#### 2.1.1. Immunobullous Skin Diseases

Immunobullous diseases represent a heterogeneous group of entities in which the typical lesion is a blister due to the loss of adhesion between intraepidermal cells or between the basal keratinocytes to the basement membrane; according to the degree of systemic repercussions, these diseases may require critical treatment, with pemphigus vulgaris as its potentially most serious form. The bullous pemphigoid, more common in the elderly, usually has a self-limited course with low mortality rate.

The paraneoplastic pemphigus (PNP) is a variety of pemphigus with differential characteristics. The PNP is an autoimmune blistering syndrome that can affect multiple organs other than skin and is closely related with benign or malignant tumours especially lymphomatoid and hematologic malignances. Patients with PNP can develop life-threatening restrictive bronchiolitis obliterans that is irreversible with lung transplant being the only possible alternative. PNP treatment should be focused on the eradication of the associated tumour process. Cutaneous manifestations usually have a poor response to treatment, being better than those associated with benign processes such as thymoma or Castleman's disease [22].

The pemphigus group has desmogleins as the main antigen. Autoantibodies directed against these proteins inhibit their adhesive function, causing loss of the intercellular binding of keratinocytes.

The pemphigus group of blistering autoimmune diseases is characterized by mucocutaneous blisters that break easily and become erosions. Upon examination, a positive Nikolsky's sign (skin detachment when applying lateral pressure on the skin near an ampoule) is typical, and the diagnosis is made by skin biopsy. Treatment should combine skin care with anti-inflammatory and immunosuppressive aetiological treatment with corticosteroids, immunoglobulins, and, in recent years, mycophenolate mofetil; biological therapies such as rituximab have been added with satisfactory results [23].

#### 2.1.2. Generalized Pustular Psoriasis

Generalized pustular psoriasis (GPP), also known as Von Zumbusch disease, is an inflammatory skin disorder with multisystemic and potentially fatal affectation. Recently, a genetic mutation in *IL36RN* (key component of the innate immune system) has been suggested as the basis of generalized pustular disease [24, 25]. It is characterized by the appearance of erythematous plaques with sterile pustular lesions followed by extensive desquamative areas and associated with a systemic inflammatory response syndrome. The severity of the condition usually requires systemic treatment such as methotrexate or ciclosporin. The best pathophysiological knowledge of the disease justifies the use of new therapies with the ability to block tumour necrosis factor *α* or other involved interleukins [26, 27].

#### 2.1.3. Erythroderma

Erythroderma is an exfoliative dermatitis characterized by erythema that affects more than 90% of the skin surface with a variable degree of desquamation. It is a cutaneous manifestation of various skin diseases (psoriasis and atopic dermatitis), drug reactions (allopurinol, carbamazepine, ciprofloxacin, etc.), or malignant processes (T-lymphoma or paraneoplastic phenomena) [28]. Patchy areas of erythroderma are observed and evolve to affect most of the skin surface accompanied by a variable degree of scaling. The skin appears as intense red, bright, dry, and scaly and is often accompanied by pruritus and ectropion. Frequently, the clinical presentation indicates the underlying disease, with psoriasis being the most frequent cause followed by drug reactions [29]. Skin biopsy may be useful to determine the aetiology, although sometimes the findings are nonspecific and may require multiple biopsies. Erythroderma treatment is based on controlling the underlying disease, removing external contributing factors, and providing supportive therapy. At the systemic level, adequate hydration must be maintained with fluid replacement, electrolyte correction, and meticulous skin care [30].

#### 2.1.4. Severe Cutaneous Adverse Reactions (SCARs)

SCARs are unpredictable and infrequent but have high morbidity and mortality. Adverse drug reactions alter the immune response and cause extensive epidermolysis. The main conditions that encompass noninfectious SCARs are Stevens–Johnson syndrome (SJS), toxic epidermal necrolysis (TEN), and drug-induced hypersensitivity syndrome/drug reaction with eosinophilia and systemic symptoms (DISH-DRESS) [31].

SJS and TEN are considered the same entity, with different degrees of severity. They are characterized by erythema with extensive epidermal detachment and mucous membrane erosion. The classification is based on the percentage of affected skin area. Epidermolysis of less than 10% is defined as SJS, detachment between 10 and 30% is classified as SJS/TEN overlap, and detachment greater than 30% is classified as TEN [32].

The first clinical manifestation is usually fever and malaise followed by generalized erythema with the appearance of blisters that break easily and erosion in mucous membranes of the mouth, eyes, and genital area. In the most extensive forms, erosion and necrosis can affect the conjunctiva, bronchi, trachea, intestine, and kidney, with a mortality ranging between 25 and 30%. The severity of the clinical picture can be determined using the SCORTEN severity scale, designed as a predictor of mortality from toxic epidermal necrolysis. It uses 7 easy-to-measure items: age, presence of malignancy, tachycardia, degree of skin detachment, and plasma levels of urea, blood glucose, and bicarbonate [33].

**Figure 1:**
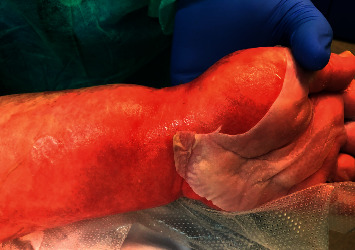
Toxic epidermal necrolysis (TEN): detachment of the top layer of skin of the upper extremity.

Some medications alter the immune response, together with a genetic susceptibility that causes the development of SCARs [32]. Sulfonamides are the most frequently implicated drugs, followed by cephalosporins, quinolones, imidazoles, and anticonvulsants such as carbamazepine and lamotrigine (Figure 1). In recent years, specific human leucocyte antigen (HLA) genotypes have been associated with the appearance of SCARs with the use of carbamazepine [34].

DISH-DRESS is a drug-induced, multisystemic, and infrequent reaction that occurs between 3 and 6 weeks after the administration of a drug and has been associated with the reactivation of herpes virus 6. The main causative agents are anticonvulsants (carbamazepine, phenytoin, phenobarbital, mexiletine, and lamotrigine), allopurinol, and sulfasalazine. It is defined by a generalized, long-lasting maculopapular rash or any other skin rash that progresses occasionally to erythroderma, accompanied by fever, lymphadenopathy, and impaired liver function as the most common organ involvement [35].

Diagnosis by skin biopsy is the only method to confirm the nature of the skin reaction [36]. The main differentiating diagnosis is scalded skin syndrome caused by *Staphylococcus* species and other cutaneous autoimmune skin diseases such as pemphigus vulgaris and pemphigoid of the mucous membrane. Treatment should include immediate withdrawal of the suspect drug and life-support therapy [37].

#### 2.1.5. Toxic Shock Syndrome

Toxic shock syndrome (TSS) is a rare, acute Gram positive infection characterized by fever, malaise, gastrointestinal symptoms, confusion, weakness, and refractory hypotension associated with diffuse, red, macular rash that can involve both skin and mucous membranes, conjunctival hyperaemia, and “strawberry tongue.” Desquamation occurs 1-2 weeks after disease onset. TSS is typically caused by exotoxins produced by *Staphylococcus aureus* or group A *Streptococcus* [38]. Some cases have been related to the use of vaginal tampons and vaginal colonization by *Staphylococcus* facilitating the production of exotoxin. Nonmenstrual cases may result around surgical site, burns, nasal packing, postinfluenza pneumonia, postpartum infections, or insulin pump infusion sites, or no source may be identified. Patients should be managed in accordance with sepsis guidelines. Antibiotics should be initiated against *S. aureus* and *S. pyogenes* and an aggressive source control is mandatory, which may include surgical debridement, removal of invasive devices, or vaginal examination in the case of suspected menstrual TSS [39].

#### 2.1.6. Staphylococcal Scalded Skin Syndrome

Staphylococcal scalded skin syndrome (SSSS) is a bullous disease that is potentially life-threatening due to infection with *Staphylococcus aureus*, which produces an exfoliative exotoxin [40]. The bacterium is located in mucous membranes, and its toxin spreads through the bloodstream, causing intraepidermal detachment due to breakage of the desmoglein 1 complex far from the initial focus (positive Nikolsky's sign). This pathology can affect any age, but it is more frequent in children under 5 years of age; however, when it affects adults, it is usually more serious, with mortality up to 60%. It can be confused with TEN, but its main differences are that it does not usually affect mucous membranes, is not related to drug exposure, and has a different histological pattern. In the treatment, the initiation of the appropriate antibiotic drug early is essential [41].

### 2.2. Dermatological Disorders Associated with Systemic Diseases

DMs can precede, accompany, or follow the evolution of a systemic disease. A wide variety of diseases can present DMs. The DMs that we can most frequently find in the field of intensive care are secondary to peripheral vascular damage produced in the context of a systemic underlying disease or are manifestations of an immune disease such as vasculitis or connective tissue diseases.

#### 2.2.1. Purpura Fulminans

Venous insufficiency with cutaneous infarction and disseminated intravascular coagulation can appear in the context of severe sepsis; however, it can also be due to a congenital deficit of protein C or protein S. Classically, it has been related to the most serious forms of meningococcal sepsis, but it can also be present in sepsis due to *Haemophilus*, *Streptococcus*, *Staphylococcus*, or less frequent germs such as *Rickettsia*, especially in anaesthetic patients [42–44].

**Figure 2:**
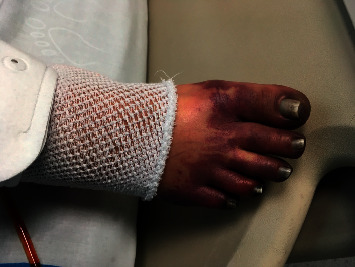
Purpura fulminans: haemorrhagic necrosis of the skin in the patient's foot.

The characteristic lesions are very well-delimited petechiae or ecchymoses, usually located on the trunk and extremities that can progress to extensive areas of cutaneous necrosis, especially in acral areas; development is favoured by the simultaneous administration of vasopressor drugs (Figure 2). Histology shows endothelial damage with areas of thrombosis in the vascular wall [45]. Skin lesion biopsy rarely reveals the bacteria responsible for the disease. Treatment is aimed at controlling the septic process.

#### 2.2.2. Cutaneous Lesions due to Direct Vascular Injury

**Figure 3:**
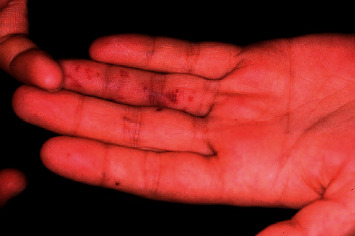
Janeway lesions: erythematous macular painless rashes distributed along the tips of the fingers.

Some systemic infectious processes present cutaneous manifestations due to direct vascular injury such as ecthyma gangrenosum, Janeway spots, or Osler nodules. The gangrenous ecthyma, related to systemic infection by *Pseudomonas aeruginosa*, manifests as a maculopapular lesion with a central vesicle that ruptures and leaves an ulcer with a painful necrotic bottom. Osler nodules and Janeway spots are produced by septic microemboli in the context of bacterial endocarditis. Osler's nodules are small, reddish, painful, raised lesions that appear on the thumb, fingers, and toes; Janeway's spots, on the other hand, are small haemorrhagic, nonpainful lesions located on palms and soles (Figure 3). Treatment is directed to the germ responsible for the septic process [46].

#### 2.2.3. Calciphylaxis

Calciphylaxis is a rare multifactorial cutaneous vascular disease with predilection for patients with end-stage renal disease characterized by occlusion of microvessels in the subcutaneous adipose tissue and dermis. Calciphylaxis causes painful cutaneous lesions and is frequently accompanied by tactile hyperesthesia. The skin manifestations include necrotic ulcers, livedo racemosa, haemorrhagic patches, haemorrhagic bullae, and indurated plaques. A dusky discoloration of the skin indicates and area of imminent necrosis [47]. Sepsis originating from these resultant wounds is considered the most common cause of death. Although skin manifestations dominate the clinical presentation, calciphylaxis is likely a systemic disorder and extraskeletal calcifications are often found on imaging studies. Skin biopsy is the standard method for confirmation suspected calciphylaxis. However, the biopsy is not needed in patients with typical lesions and end-stage renal disease. Treatment should be aimed at controlling pain and wound care with removal of necrotic tissue. Other therapeutic options such as sodium thiosulfate or bisphosphonates have proven effective in some cases [48].

#### 2.2.4. Cutaneous Vasculitis

Vasculitis is a group of complex and disabling diseases that are characterized by an inflammatory infiltrate in or around blood vessels that causes vascular damage. Skin involvement is common, and its form of presentation depends on the size of the affected vessel. The classification of vasculitis, much debated in recent years, is based on the size of the affected vessel, the pathogenesis, and the main organ involved [49]. We distinguish 2 types of cutaneous vasculitis: small vessel vasculitis and single-organ vasculitis. Clinically, the typical lesion is palpable purpura; however, other lesions such as ulcers, blisters, or livedo reticularis may be associated. Palpable purpura is very suggestive of small vessel vasculitis and affects arterioles, capillaries, and postcapillary venules. Skin biopsy is the gold diagnostic standard and allows differentiation from other nonvasculitic processes with similar clinical manifestation due to vessel wall dysfunction or their occlusion, as observed in coagulation diseases or purpura fulminans.

The most typical presentation is leukocytoclastic vasculitis or small vessel vasculitis restricted to the skin. The most frequent manifestation is palpable purpura (approximately 2–10 mm in diameter) located in the lower extremities and buttocks with a symmetrical distribution. On occasion, arthralgias and/or arthritis may also appear with general symptoms such as fever, malaise, anorexia, and myalgias. Systemic involvement is rare, and if it occurs, it is of mild intensity [50].

The treatment of this vasculitic process is symptomatic. In cases with extensive skin involvement, the use of corticosteroids may be effective. Severe forms may require corticoids at higher doses, dapsone, and even azathioprine or rituximab [51].

Another type of small vessel vasculitis is ANCA-associated vasculitis (AAV) that includes granulomatosis with polyangiitis, microscopic polyangiitis (MPA), and eosinophilic granulomatosis with polyangiitis (EGPA). AAV can affect multiple organs systems and the patient may need to be admitted to the ICU. Granulomatosis with polyangiitis, previously known as Wegener's granulomatosis, is defined as a necrotizing granulomatous inflammation of the upper and lower respiratory tracts with necrotizing vasculitis of small and medium-size vessels and usual renal involvement. MPA belongs to the systemic vasculitis and the major organs involved are the kidneys and the lungs. EGPA, previously called Churg–Strauss syndrome, typically occurs in patients with preexisting asthma and involves the skin, lungs, heart, and peripheral nerves.

The most common skin manifestation of ANCA-associated vasculitis is palpable purpura located on limbs. Other manifestations include livedo reticularis, nodules, urticarial lesions, and skin ulcers with necrosis [52].

Cutaneous manifestations in vasculitis are not exclusive to those affecting the small vessel. Medium and large vessel vasculitis can show DMs even though the affected vessels are not in the skin [53]. In large vessel vasculitis such as Takayasu arteritis and giant-cell arteritis, Raynaud's phenomenon, digital gangrene, or unilateral digital clubbing, is typical, although in Europe and North America it is more frequent to manifest as acute inflammatory nodules or erythema nodosum-like lesions. Two major types of medium vessel vasculitis are Kawasaki disease and polyarteritis nodosa (PAN). Kawasaki disease is characterized by a mucocutaneous lymph node and polymorphous exanthema varying from macular to maculopapular or morbilliform. The most frequent DMs of PAN are, in the order of frequency, purpura, livedo, and nodules with distal gangrene being less frequent. The systemic form of PAN is the most severe and 28 to 60% are accompanied by skin manifestations. The cutaneous form of PAN, less frequent, may present nodules (74–80%), livedo reticularis (56–74%), and ulcerations (8–51%) [54].

#### 2.2.5. Connective Tissue Diseases

Connective tissue diseases such as scleroderma, lupus erythematosus, and dermatomyositis within the group of idiopathic inflammatory myopathies are autoimmune diseases of unknown origin that can affect different organs and have characteristic DMs. These 3 pathologies may require admission to the ICU due to their impact on vital organs or complications derived from immunosuppressive treatment. However, some patients may present diagnostic criteria for more than one specific connective tissue disease, as in mixed connective tissue disease (MCTD), making specific diagnosis difficult.

Scleroderma is characterized by the thickening and adhesion of the tegument due to increased production of collagen in the dermis, hypodermis, and some organs which leads to fibrosis of the skin and underlying tissues. Localized or morphea forms exist, and systemic forms can include organ involvement.

Systemic lupus erythematosus (SLE) can affect any organ and tissue of the body. At the cutaneous level, the most typical manifestation is a malar rash or butterfly rash characterized by raised erythematous lesions at the malar level, along the nasolabial sulcus, that can be precipitated by sun exposure and leaves no scar. Moreover, skin lesions could be plaques with epidermal atrophy that can leave a hypopigmented area on sun-exposed and sun-protected areas [55].

The main manifestations of dermatomyositis are skin disorders and skeletal muscle weakness. One of the most typical lesions is Gottron's papules, present in more than 70% of cases, in the form of slightly raised lesions with a purplish appearance that appear on the back of the metacarpophalangeal and interphalangeal joints. Also characteristic is Gottron's sign with macules or erythematous plaques that are located on the back of joints such as the elbow or knee. Other common lesions are heliotrope erythema in the form of violet-coloured or periorbital erythematous macules, periungual telangiectasias, and symmetrical violaceous erythema, which appear in exposed areas such the front and back of the upper trunk, arms, or face [56]. The recognition of these DMs is of special interest due to their association in more than 30% of cases with neoplasms, interstitial pulmonary fibrosis, and heart disease. Autoantibodies in idiopathic inflammatory myopathies research will help us identify different clinical subgroups [57, 58].

MCTD is a rare systemic autoimmune rheumatic disease characterized by the association of clinical manifestations of SLE, systemic skin sclerosis, and dermatomyositis in the presence of high levels of anti-U1-RNP antibodies. Symptoms at the cutaneous level are varied [59]. Raynaud's phenomenon is an early feature in more than 90% of patients. Sclerodactyly and nail fold vascular changes are also common. Skin rashes may resemble lupus or dermatomyositis. Digital infarcts may present in some patients and it is rare that it presents as truncal scleroderma. These symptoms are usually accompanied by polyarthritis, hand oedema, myositis, and esophageal hypomotility. Treatment is based at controlling symptoms and should be early in the face of potentially serious manifestations such as pulmonary hypertension. Immunosuppressive therapy and steroids remain the therapeutic mainstay for MCTD [60].

### 2.3. Dermatological Manifestations Secondary to the Situation and Management of Critical Patients

Most DMs appear during admission to the ICU and are due to factors surrounding critical patients. During the stay in the ICU, immobilization, malnutrition, impaired tissue perfusion, immune dysfunction, fluctuations in body temperature, or difficulties in maintaining hygiene are factors that affect the cutaneous barrier and favour the appearance of cutaneous lesions [20]. We include lesions of infectious origin, from drug reactions or secondary to the use of devices.

#### 2.3.1. Dermatological Disorders of Infectious Origin

Lesions secondary to an infectious agent are the most frequent DMs in the intensive setting and appear in the evolution of critical illness [61]. Bedding, humidity, obesity, etc., favour the appearance of inflammatory dermatitis in large skin folds, with stratum corneum damage favouring the entry of germs such as *Candida albicans* or beta-haemolytic *Streptococcus* bacteria.

**Figure 4:**
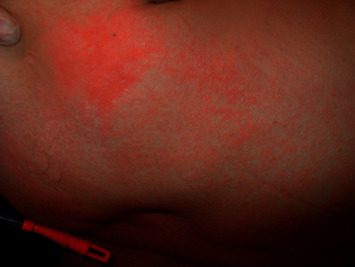
Morbilliform rash: maculopapular exanthem on the trunk.

Superficial mycosis caused by *Candida* is the most common problem. It starts at the bottom of the fold as an erythematous plaque that is wet and bright red and well-delimited. Furrowing of the fold and the presence of papulopustular lesions outside the erythematous plaque (satellite lesions) are characteristic (Figure 4). The diagnosis is usually clinical and can be confirmed by scraping the lesions followed by direct microscopic examination with potassium hydroxide stain or by culture. Local treatment with antifungal agents is usually sufficient, in addition to the establishment of preventive measures with the routine use of skin protectants to keep the skin clean, dry, and hydrated [62].

#### 2.3.2. Dermatological Disorders from Drug Reactions

**Figure 5:**
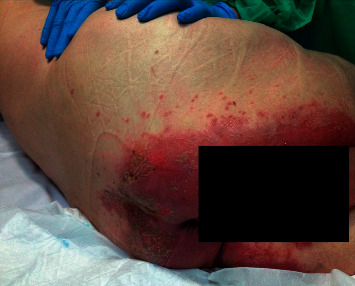
Candida intertrigo: genitocrural intertrigo in the inframammary fold infected by *Candida albicans*. *Note.* Satellite lesions.

Cutaneous eruptions resulting from drug reactions in the intensive field occur in 10% of cases. The complexity of critical patients, usually polytreated and with a context of immunosuppression, favours the appearance of adverse drug reactions at the cutaneous level. The most frequent form of presentation is a morbilliform rash characterized by erythematous macules and papules, usually with symmetric distribution, which may be confluent in plaques. They predominate in the trunk and pressure areas and extend to the extremities and face may be spared (Figure 5). This morbilliform eruption usually appears approximately 1 week after starting the responsible drug and lasts between 1 or 2 weeks. The most common drugs associated with rash are penicillins and derivatives, sulfonamides, and anticonvulsants [63].

Urticaria is another rash secondary to drugs. The rash appears as raised lesions with a paler central area and an erythematous halo that are produced by oedema of the superficial dermis; urticaria is accompanied by itching. The lesions appear within the first 24 hours after exposure to the responsible drug and resolve in the next 24–48 hours. Approximately 20% of urticaria cases are accompanied by angioedema consisting of oedema of the mucous membranes, including the respiratory and intestinal tracts. The drugs commonly associated are penicillins and derivatives and nonsteroidal anti-inflammatory drugs.

Treatment is based on the withdrawal of the suspect drug and antihistamines or steroids for symptom control [36].

#### 2.3.3. Dermatological Disorders Secondary to Devices

Patients admitted to the ICU require advanced monitoring and treatment that require the use of various devices, both invasive and noninvasive, that can cause varying degrees of DMs. Complications from the use of devices have usually been related to their capacity to produce infection [64] or to problems related to their placement [65, 66]. Invasive procedures or some devices used in the monitoring or treatment of critical patients can cause iatrogenic injuries or inflammatory reactions that cause contact dermatitis [8].

When implanting a catheter or performing a tracheostomy, for example, the skin barrier is broken, which can cause injuries that become permanent; sometimes the injuries are imperceptible, but sometimes they leave an unsightly lesion.

Contact dermatitis is an eczematous disease due to the inflammatory reaction of the skin to an external agent such as antiseptics, self-adhesive electrodes, or other devices [67].

#### 2.3.4. Dermatological Disorders-Related Pressure Injury

Critically ill patients are at high risk of developing pressure ulcers. Factors related to the development of these ulcers are prolonged stay, immobility, deep sedation, use of vasoactive drugs, hypotension, anasarca, organ dysfunction, and malnutrition [68]. There are various strategies aimed at preventing the appearance of ulcers such as the use of pressure redistribution equipment, intense physiotherapy, protective dressings, and trying to achieve an optimal state of general health. However, despite use of best practice measures, pressure ulcers can occur, and their identification and early treatment are essential for their good evolution [69].

#### 2.3.5. Dermatological Disorders Induced by Vasopressor Drugs

Skin necrosis can appear as a complication of the infusion of vasopressor drugs. Classically, it is related to the use of high doses of dopamine and norepinephrine. The existence of disseminated intravascular coagulation and cardiocirculatory shock are risk factors for the development of ischemic skins lesions that can progress to symmetrical peripheral gangrene related to vasopressors [70]. Vasopressin, useful in the treatment of catecholamine-resistant vasodilatory shock, may also provoke cutaneous ischemia, due to its vasoconstrictor action on the arteriolar level. The ischemic skin lesions are normally localized in the distal area of the extremities and the trunk. When the administration of vasopressin is by a peripheral vein, it may provoke local cutaneous ischemia if it infiltrates into the subcutaneous tissue. To avoid the potential for ischemic lesions, administration of vasopressin should be by central venous catheter, and careful monitoring of the extremities to detect ischemic changes should be carried out. Early recognition and prompt treatment with systemic, local, or topical sympathetic blockers or vasodilators may be beneficial in treating ischemia and the definitive treatment that has been established for gangrene is amputation of ischemic limbs [71].

### 2.4. Previous Dermatological Disorders

**Figure 6:**
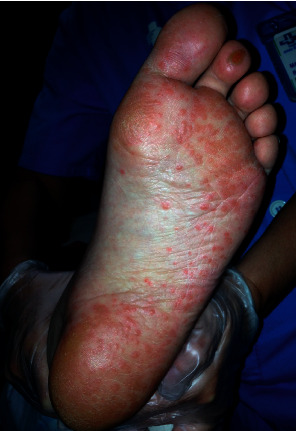
Secondary syphilis: maculopapular lesions on bottom of the foot.

Patients may present dermatological disorders that may need to be identified when admitted to the ICU (e.g., when a patient cannot provide his or her medical history or information is not available). Some chronic skin diseases such as psoriasis or connective tissue diseases must be recognized and could be exacerbated during admission, requiring specific treatment. Other processes, such as lichen planus and atopic eczema, may require symptomatic treatment. Other diseases, due to their characteristics, may involve chronic treatment that must be taken into account in the management of the admitted patient, or the patient may manifest lesions unrelated to the pathology of admission that do not require treatment but that may complicate the evaluation of the patient, such as cutaneous tumours, cutaneous infectious diseases, and pigmentation disorders; in these cases, it is necessary to consult a dermatology specialist (Figure 6).

## 3. Conclusions

The present review includes the DMs that may appear in the intensive care setting. In many cases, making it difficult to diagnose, the patient cannot manifest clinical aspects (pain, itching, etc.) because of sedation or neurological disorders.

The algorithm proposed by Jack et al. can be used to identify the most frequent DMs in ICU. In this algorithm, DMs with vital compromise are first identified. In the next step, differentiate the processes that appear during or before ICU admission and determine the priorities of the consultation with the dermatology specialist [72].

The great variety of skin problems highlights the importance of including a systematic examination of the skin in ICU care routines [73].The classification we have used defines different groups of skin pathologies.

Life-threatening DMs are infrequent, but they must be quickly recognized, and initiation of specific treatment and maintenance at an early stage is essential. DMs associated with systemic diseases offer the possibility of adding dermatological signs to skin examinations that help in the diagnosis of underlying disease. In addition, DMs that occur during the management of critical patients must be expected and diagnosed during daily patient care.

The complexity of critical patients requires the collaboration of different health professionals. Intensive care professionals should consult dermatologists. We must provide comprehensive care covering any medical and surgical pathology; therefore, detection and control of different skin problems should be incorporated into routine quality care [74].

## Figures and Tables

**Table 1:** Classification of dermatological manifestations in critical patients.

Classification of dermatological manifestations in critical patients
*1. Dermatological manifestations of life-threatening skin diseases*
1.1. Immune-bullous diseases
1.2. Generalized pustular psoriasis
1.3. Erythroderma
*2. Severe cutaneous adverse drug reactions*
2.1. Toxic shock syndrome
2.2. Staphylococcal scalded skin syndrome
*3. Dermatological manifestations of underlying systemic diseases*
3.1. Purpura fulminans
3.2. Cutaneous lesions caused by direct vascular injury
3.3. Calciphylaxis
3.4. Cutaneous vasculitis
3.5. Connective tissue diseases
*4. Dermatological manifestations as a consequence critical illness*
4.1. Dermatological disorders of infectious origin
4.2. Dermatological disorders from drug reactions
4.3. Dermatological disorders secondary to devices
4.4. Dermatological disorders related pressure injuries
4.5. Dermatological disorders induced by vasopressor drugs
*5. Previous dermatological manifestations*

**Table 2:** Published studies on dermatological disorders in critical patients.

	Dunnill et al.	Badia et al.	George et al.	Henk et al.	Prashanth and Pai	Wollina and Nowak	Badia et al.	Emre et al.	Agrawal et al.	Chang et al.	Pektas and Demir	Pektas and Demir	Awal and Kaur	Gupta et al.
Publication year	1994	1999	2008	2011	2012	2012	2013	2013	2013	2014	2017	2017	2018	2018
Period (years)	1	2	11	3	2	1	3	1	3	1	3	3	2	<1
Country	UK	Spain	UK	Holland	India	Deutschland	Spain	Turkey	India	Guatemala	Turkey	Turkey	India	India
Type of study	Retrosp.	Prosp.	Retrosp.	Prosp.	Retrosp.	Retrosp.	Prosp.	Prosp.	Prosp.	Prosp.	Prosp.	Prosp.	Prosp.	Prosp.
	UNI	UNI	MULTI	UNI	UNI	UNI	UNI	UNI	UNI	UNI	UNI	UNI	UNI	UNI
Type of patient	Adult	Adult	Adult	Pedia.	Pedia.	Adult	Adult	Adult	Adult	Adult	Adult	Adult	Adult	Mix
No. of patients	27	46	2406	42	318	55	133	82	427	47	151	73	312	50
No. of lesions	27	51	2406	46	361		154	90	427	47		73	312	50
Type of lesions (N)														
Life-threatening	5	0	199	3	3	7	5	—						
Systemic	10	14	61	18	284	—	26	—	29	—	—	0	20	2
Associate	9	34	12	15	8	20	45	61	159	38	—	0	109	1
Previous	3	3	191	2	14	8	—	—	67	—	96	60	121	36
Other	—	—	1943	8	9	20	14	29	94	—	13	2	46	1
Acute skin failure (N)			199				5	1	10				9	
APACHE II			17.2				18		17.4					
Length of stay (IQR)		19	—			16	23	5–57			10 (13)			17 (3–43)
Mortality ICU (%)	33	26	27.5	16		21.8			46.1		31.1	46.6	11.2	
Mortality HOSP (%)			39.6				38.3							

Prosp.: prospective study. Retrosp.: retrospective study. UNI: unicenter study. MULTI: multicenter study.

## Data Availability

No data were used to support this study.

## Abbreviations

AAV: ANCA-associated vasculitis
DMs: Dermatological manifestations
DISH-DRESS: Drug-induced hypersensitivity syndrome/drug reaction with eosinophilia and systemic symptoms
EGPA: Eosinophilic granulomatosis with polyangiitis
GGP: Generalized pustular psoriasis
ICU: Intensive care unit
MPA: Microscopic polyangiitis
MCTD: Mixed connective tissue disease
MMP: Mucous membrane pemphigoid
PAN: Polyarteritis nodosa
PNP: Paraneoplastic pemphigus
SCARs: Severe cutaneous adverse reactions
SJS: Stevens–Johnson syndrome
SLE: Systemic lupus erythematosus
SSSS: Staphylococcal scalded skin syndrome
TSS: Toxic shock syndrome
TEN: Toxic epidermal necrolysis.

## Additional Points

DMs in critical patients are relatively frequent, and they are predominantly consequent to critical illness, treatment, or marker of extracutaneous pathology. The great variety of skin problems highlights the importance of including a systematic examination of the skin in ICU routine quality care.
